# Liver Transplant Recipient Characteristics Associated With Worse Post-Transplant Outcomes in Using Elderly Donors

**DOI:** 10.3389/ti.2022.10489

**Published:** 2022-08-25

**Authors:** Shingo Shimada, Tayseer Shamaa, Tommy Ivanics, Toshihiro Kitajima, Kelly Collins, Michael Rizzari, Atsushi Yoshida, Marwan Abouljoud, Dilip Moonka, Mei Lu, Shunji Nagai

**Affiliations:** ^1^ Division of Transplant and Hepatobiliary Surgery, Henry Ford Health System, Detroit, MI, United States; ^2^ Division of Gastroenterology and Hepatology, Henry Ford Health System, Detroit, MI, United States; ^3^ Department of Public Health Sciences, Henry Ford Health System, Detroit, MI, United States

**Keywords:** liver transplantation, elderly donors, patient characteristics, posttransplant outcome, organ procurement and transplant network and united network for organ sharing

## Abstract

Advanced age of liver donor is a risk factor for graft loss after transplant. We sought to identify recipient characteristics associated with negative post-liver transplant (LT) outcomes in the context of elderly donors. Using 2014–2019 OPTN/UNOS data, LT recipients were classified by donor age: ≥70, 40–69, and <40 years. Recipient risk factors for one-year graft loss were identified and created a risk stratification system and validated it using 2020 OPTN/UNOS data set. At transplant, significant recipient risk factors for one-year graft loss were: previous liver transplant (adjusted hazard ratio [aHR] 4.37, 95%CI 1.98–9.65); mechanical ventilation (aHR 4.28, 95%CI 1.95–9.43); portal thrombus (aHR 1.87, 95%CI 1.26–2.77); serum sodium <125 mEq/L (aHR 2.88, 95%CI 1.34–6.20); and Karnofsky score 10–30% (aHR 2.03, 95%CI 1.13–3.65), 40–60% (aHR 1.65, 95%CI 1.08–2.51). Using those risk factors and multiplying HRs, recipients were divided into low-risk (*n* = 931) and high-risk (*n* = 294). Adjusted risk of one-year graft loss in the low-risk recipient group was similar to that of patients with younger donors; results were consistent using validation dataset. Our results show that a system of careful recipient selection can reduce the risks of graft loss associated with older donor age.

## Graphical Abstract

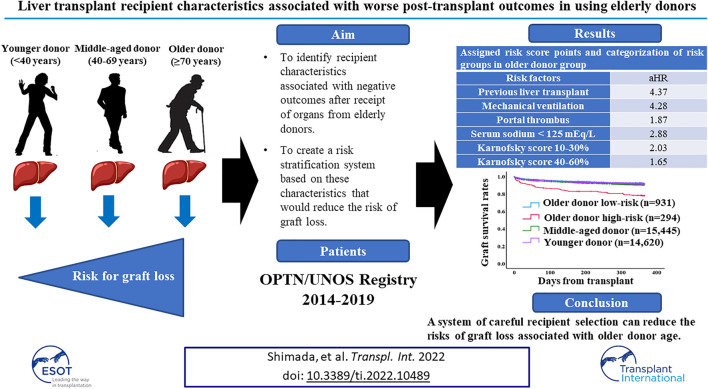

## Introduction

The need for donor livers currently exceeds the number of organs available for transplantation in the US [1]. For example, in 2019 there were 12,767 new registrations for liver transplantation (LT), but only 8,896 were performed [2]—underscoring the importance of expanding the donor pool. However, expanding the donor pool by using older donors may compromise post-LT outcomes. Higher donor age is a significant risk factor for graft loss and mortality after LT [3, 4] and for ischemia-reperfusion injury, with increased necrosis and apoptosis [5, 6]. Although a donor age of ≥70 years is considered the highest risk category [3], by 2030 the proportion of the US population older than 70 will have increased from 9% to almost 14% [7]. Within this context, optimizing the usage of grafts from older donors is essential.

Previous studies have investigated recipient risk factors for poor liver transplantation outcomes when using livers from older donors [8-10]; these include previous LT or abdominal surgery, active hepatitis C virus (HCV) infection, and hepatocellular carcinoma (HCC), as well as current hospitalization, need for pre-transplant dialysis, and registration as status 1 (risk of imminent demise) [8–10]. However, given the rising age of both donors and recipients, the introduction of highly effective direct-acting antiviral treatments for HCV, and changes in liver allocation policy, a more current appraisal of factors associated with successful outcomes after liver grafts with transplantation from elderly donors is necessary.

In this study, we hypothesized that using liver from older donors could be optimized by carefully considering the medical and surgical conditions of recipients. We sought to identify recipient characteristics associated with negative outcomes after receipt of organs from elderly donors and to create a risk stratification system based on these characteristics that would reduce the risk of graft loss. The primary endpoint was set for one-year graft loss which includes patient death.

## Patients and Methods

### Study Population

**FIGURE 1:**
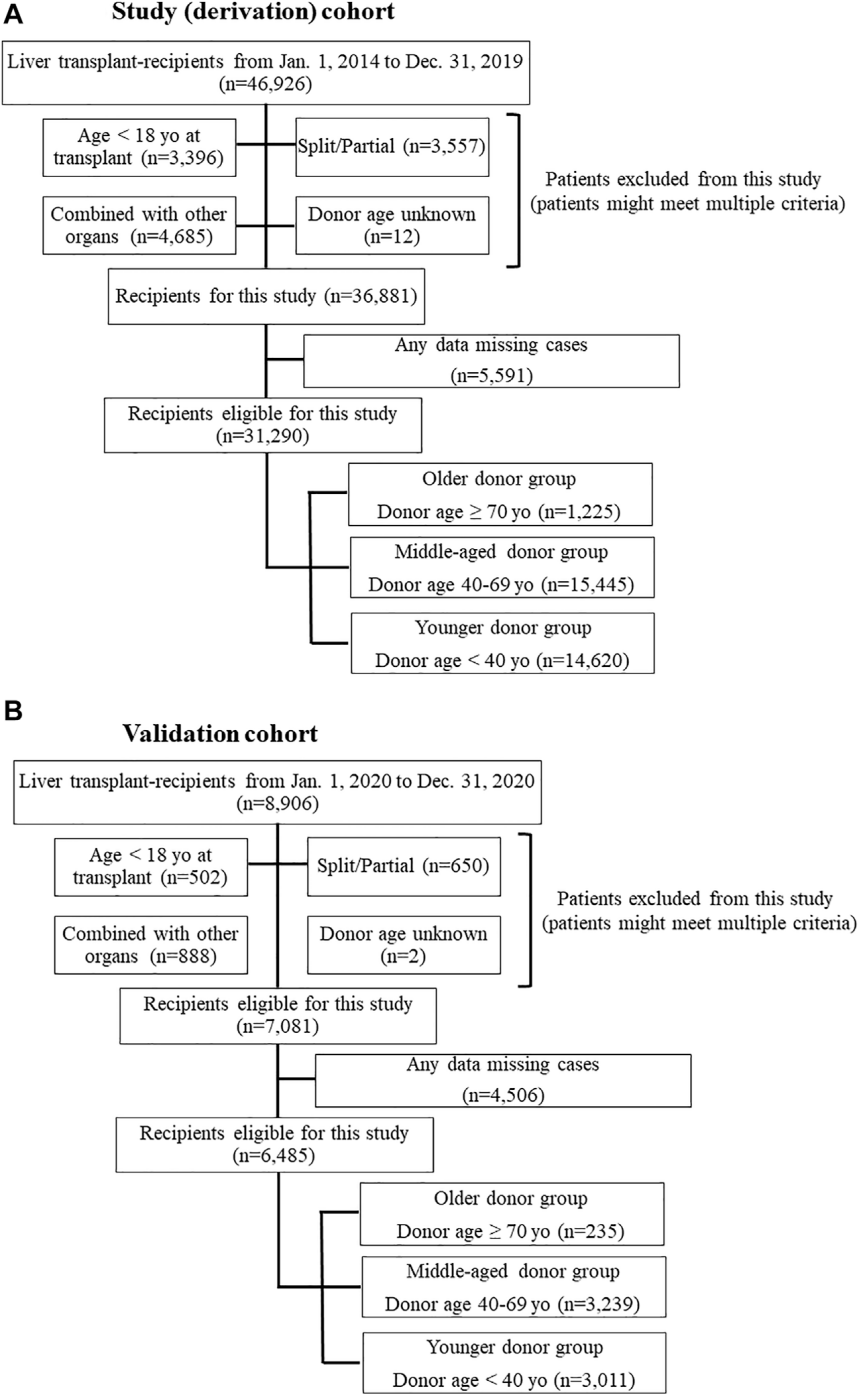
**(A)** Flow chart of study population (derivation) selection. **(B)** Flow chart of validation population selection.

This study used data from the Organ Procurement and Transplantation Network/United Network for Organ Sharing (OPTN/UNOS) Standard Transplant and Research (STAR) files for LT. The study period was set from 1 January 2014 to 31 December 2019, with 1 year of post-transplant observation for each patient. Study procedures were approved by the Henry Ford Health System Institutional Review Board; the requirement for written informed consent was waived due to the deidentified nature of the data. Patients who were 18 years or older at the time of transplant were eligible for this study. Patients who received a partial/split graft or combined organ transplant with thoracic organs, kidney, intestine, and/or pancreas or patients for whom donor age was unknown were excluded. Also, if patients who had one or more missing data which was evaluated in this study, those were excluded (Figure 1). To assess the impact of donor age on post-LT outcomes and to determine whether specific recipient characteristics were associated with worse post-LT outcomes with liver grafts from older donors, the cohort was divided into three groups according to the donor age. Age categories were determined using the liver donor risk index [3]: older donor (≥70 years); middle-aged donor (40–69 years); and younger donor (<40 years).

### Covariates

Binary variables included: recipient gender; recipient diabetes; primary liver disease etiologies including HCV infection; alcohol related disease, non-alcoholic steatohepatitis and other diseases, diagnosis of HCC; history of abdominal surgery; previous liver transplant; registration as status 1; dialysis requirement at transplant; mechanical ventilation at transplant; portal thrombosis at transplant; donation after circulatory death (DCD); donor diabetes; donor history of heavy alcohol use; donor history of hypertension; and donor history of myocardial infarction. In the risk factor analysis, model for end-stage liver disease (MELD) score or MELD-sodium score was not included. Instead, 4 parameters of MELD-sodium score (serum total bilirubin, creatinine, sodium, and INR) were separately included. MELD-sodium score was calculated using the following formula; MELD-sodium = MELD +1.32 x (137-serum sodium)—[0.033 x MELD x (137 - serum sodium)] [11]. Continuous variables were classified into multilevel categorical variables. Recipient data at time of transplant included: age (<50, 50–64, and ≥65 years); BMI (<18.5, 18.5–24.9, 25.0–29.9, and ≥30.0 kg/m^2^) [12]; serum bilirubin (<2.0, [2.0–4.4, 4.5–11.9, and ≥12.0 mg/dl [total bilirubin of 2.0 mg/dl: based Child-Pugh score [13], 4.5 and 12 mg/dl were 33 and 66%tile in the cohort]); serum creatinine (<1.5, 1.5–1.7, 1.8–2.5, and ≥2.5 mg/dl [creatinine of 1.5 mg/dl is used for a diagnosis hepatorenal syndrome criteria in patients with cirrhosis] [14], 1.8 and 2.5 mg/dl were 33 and 66%tile in the cohort]), serum sodium (<125, 125–134, 135–145, and ≥146 mEq/L) [15]; and international normalized ratio (INR; <1.5, 1.5–1.7, 1.8–2.4, and ≥2.5 [INR ≥1.5; a factor of acute liver failure] [16], 1.8 and 2.5 were 33 and 66%tile in the cohort]). Organ related variables included donor age at transplantation (<40, 40–69, and ≥70 years old) and cold ischemia time (<6.0, 6.0–7.9, and ≥8 h [6 h was median value in the cohort, 8 hour-cut off point was decided according to liver donor risk index] [3]). Additional multilevel categorical variables included: recipient race (White, Black/African American, Hispanic [of any race], and other); Karnofsky Performance Status score (10–30, 40–60, and 70–100%); donor cause of death (trauma, anoxia, cerebrovascular accident [CVA], and other); and organ share type (local, regional, or national). All covariates were collected prior to or at the time of LT.

### Analysis of the Impact of Donor Age on Post-LT Outcomes

Risk of one-year graft loss after receipt of an organ from the ≥70 donor group was compared to the 40–69 and <40 donor groups. Graft loss was defined as death or re-transplantation. Analyses were adjusted for recipient demographic (age, race, gender) and clinical characteristics (BMI, diabetes, primary liver disease etiologies including HCV infection; alcohol related disease, non-alcoholic steatohepatitis and other diseases, presence of HCC, history of abdominal surgery, portal thrombus, previous liver transplant, status 1 [yes/no], laboratory values [bilirubin, creatinine, INR, sodium], Karnofsky score, and need for mechanical ventilation or dialysis) at the time of transplantation. Analyses were also adjusted for donor (age category, race, gender, BMI, diabetes, history of heavy alcohol use, history of hypertension, and history of myocardial infarction) and organ characteristics (cause of death, donation after cardiac death [DCD; yes/no], cold ischemia time, and organ share type).

### Risk Factor Analysis in the ≥70 Donor Group and Risk Stratification

**FIGURE 2:**
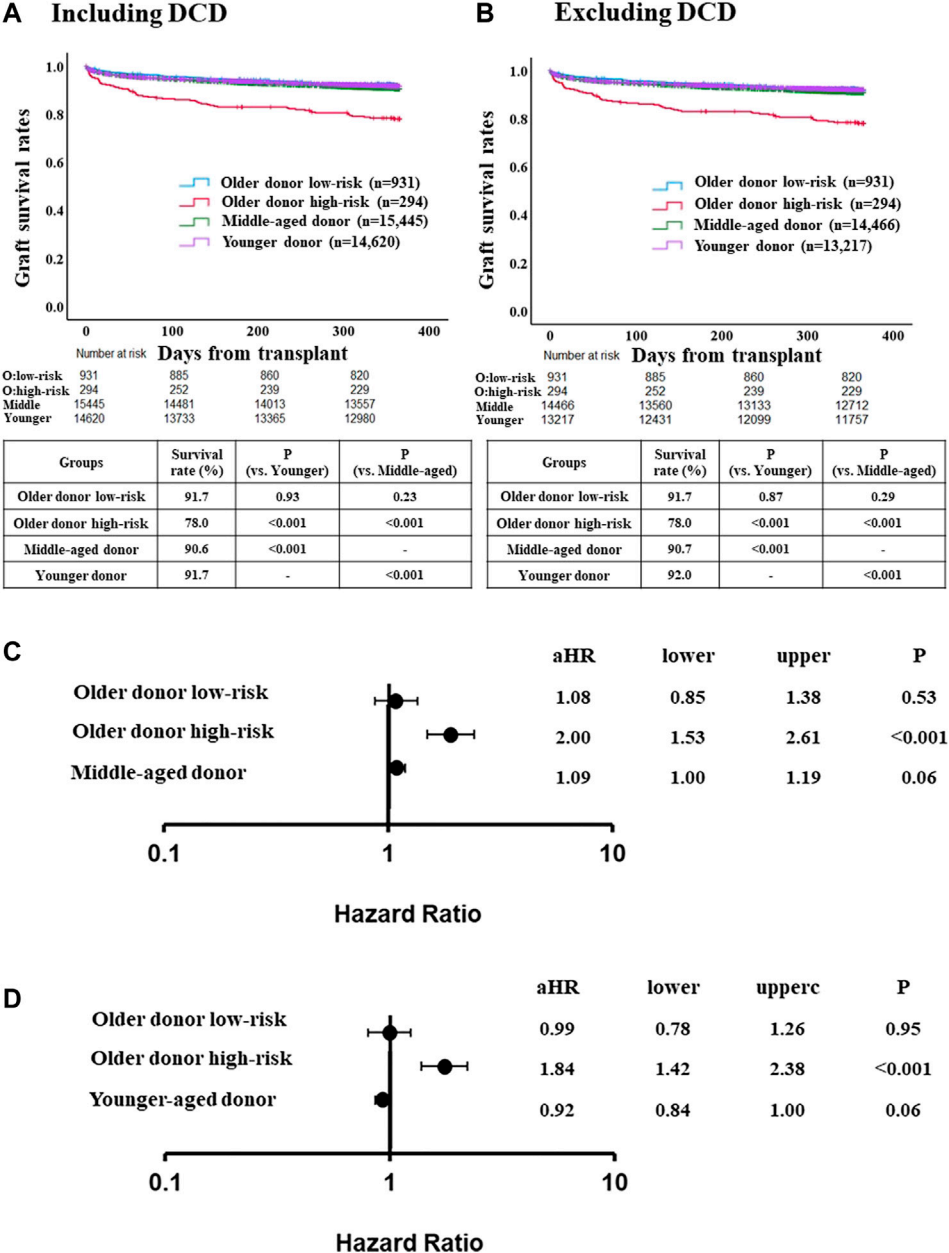
Comparison of post-LT outcome between the older donor derivation group stratified by the risk classification and middle-aged or younger donor group. **(A)** One-year graft survival rate in low-risk recipients of the older donor group was similar to those in middle-aged or younger donor group (*p* = 0.23, *p* = 0.93, respectively). **(B)** One-year graft survival rate in low-risk recipients of the older donor group was similar to those in middle-aged or younger donor group after excluding DCD cases (*p* = 0.29, *p* = 0.87, respectively). **(C)** Adjusted hazards of graft loss in the older donor derivation group stratified by the risk classification and middle-aged donor group (ref. younger donor group). **(D)** ref. middle-aged donor group. Hazards were adjusted by a multivariable Cox regression model for the following variables present at the time of transplantation: recipient age; recipient gender; recipient race; recipient body mass index (BMI); recipient diabetes; recipient primary liver disease etiologies including HCV infection, alcohol related disease, non-alcoholic steatohepatitis and other diseases; hepatocellular carcinoma; international normalized ratio; serum bilirubin; serum creatinine; Karnofsky score; history of abdominal surgery; dialysis requirement; serum sodium; portal thrombus; mechanical ventilation; previous liver transplant; status 1; cold ischemia time; donor gender; donor race; donor BMI; donor diabetes; donor cause of death; organ share type; donor history of heavy alcohol use; donor history of hypertension; and donor history of myocardial infarction.

Recipient risk factors for one-year graft loss were determined with multivariable Cox regression. The total risk score for each patient was calculated by multiplying the adjusted hazard ratios (aHRs) of recipient risk factors according to a previously used methodology [17]. If one risk factor, score is equal to the HR of that particular factor. If no risk factor, score is zero. Our risk stratification system classified recipients into low- and high-risk groups; the cut-off risk score value was calculated from Youden index and determined by the receiver operating characteristic (ROC) curve for one-year graft survival. We then compared one-year graft loss in the ≥70 donor group to these risk score categories. We also compared the low- and high-risk ≥70 donor groups to the 40–69 and <40 groups (both with and without DCD). This risk stratification system was then applied to the validation cohort using patient cohort who received LT in 2020 from the STAR files (Figure 2).

### Statistical Analysis

Patient demographic and clinical characteristics, as well as donor and organ characteristics, were described by donor age groups, using median and interquartile range (IQR) for continuous variables and number and percentages for categorical variables. We used the Mann-Whitney-U test for continuous variables and chi-square test for categorical variables to study differences in patient characteristics among the three donor age groups. Post-transplant graft survival was evaluated using Kaplan-Meier curve analysis and compared by log-rank tests. A multivariable Cox regression model assessed hazards of post-transplant graft loss. For the risk factor analysis in each donor group (older donor, middle-aged donor, and younger donor), multivariable Cox regression models were created using factors which had *p* value less than 0.157 in univariable analyses [18]. *p*-values <0.05 were considered statistically significant for all analyses. All statistical analyses were completed using SPSS version 27 (IBM, Chicago IL, United States) and R version 3.5.1 (R Foundation for Statistical Computing, Vienna, Austria).

## Results

### Patient Characteristics Among Groups

Of the 31,290 patients eligible for this study, 1,225 received livers from donors in the ≥70 group, 15,445 received livers from donors aged 40–69, and 14,620 received livers from donors <40 years old (Figure 1). Table 1 showed details characteristics of patients from the three donor age groups. Recipients of organs from older donors were themselves significantly older (median age 62 vs. 58 [donors aged 40–69] and 57 [donors <40 years], *p* < 0.001 for both).

**TABLE 1:** Comparisons of characteristics of liver transplant recipients between donor age groups.

Characteristics	Group	Older donor 70 or older	Middle-aged donor 40–69	Younger donor <40	*p* Value	*p* Value
		*n* = 1,225	*n* = 15,445	*n* = 14,620	O vs. M	O vs. Y
Median recipient age (year), [IQR]		62 [55, 66]	58 [51, 64]	57 [48, 63]	<0.001	<0.001
Recipient gender, n (%)	Male	739 (60.3)	10,404 (67.4)	9,610 (65.7)	<0.001	<0.001
	Female	486 (39.7)	5,041 (32.6)	5,010 (34.3)		
Recipient race, n (%)	White	873 (71.3)	11144 (72.2)	10,267 (70.2)	0.058	0.02
	Black	76 (6.2)	1,191 (7.7)	1,277 (8.7)		
	Hispanic	195 (15.9)	2,269 (14.7)	2,178 (14.9)		
	Others	81 (6.6)	841 (5.4)	898 (6.1)		
Recipient BMI (kg/m^2^), n (%)	18.5–24.9	326 (26.6)	3,752 (24.3)	3,926 (26.9)	0.001	0.057
	25.0–29.9	457 (37.3)	5,265 (34.1)	4,931 (33.7)		
	30.0 ≤	424 (34.6)	6,232 (40.3)	5,517 (37.7)		
	<18.5	18 (1.5)	196 (1.3)	246 (1.7)		
Median MELD (MELD-Na) score, [IQR]		18 [12, 24]	22 [14, 30]	24 [15, 34]	<0.001	<0.001
Serum bilirubin (mg/dl), n (%)	<2.0	491 (40.1)	4,773 (30.9)	4,017 (27.5)	<0.001	<0.001
	2.0–4.4	397 (32.4)	3,813 (24.7)	3,214 (22.0)		
	4.5–11.9	232 (18.9)	3,476 (22.5)	3,198 (21.9)		
	12.0 ≤	105 (8.6)	3,383 (21.9)	4,191 (28.7)		
INR, n (%)	<1.5	585 (47.8)	5,977 (38.7)	5,156 (35.3)	<0.001	<0.001
	1.5–1.7	264 (21.6)	2,791 (18.1)	2,424 (16.6)		
	1.8–2.4	264 (21.6)	3,649 (23.6)	3,606 (24.7)		
	2.5 ≤	112 (9.1)	3,028 (19.6)	3,434 (23.5)		
Serum creatinine (mg/dl), n (%)	<1.5	985 (80.4)	11,500 (74.5)	10,499 (71.8)	<0.001	<0.001
	1.5–1.7	93 (7.6)	1,207 (7.8)	1,147 (7.8)		
	1.8–2.5	98 (8.0)	1,412 (9.1)	1,466 (10.0)		
	2.6 ≤	49 (4.0)	1,326 (8.6)	1,508 (10.3)		
Serum sodium (mEq/L), n (%)	135–145	820 (66.9)	9,711 (62.9)	9,247 (63.2)	0.02	0.02
	125–134	355 (29.0)	4,943 (32.0)	4,612 (31.5)		
	<125	30 (2.4)	437 (2.8)	382 (2.6)		
	146 ≤	20 (1.6)	354 (2.3)	379 (2.6)		
History of abdominal surgery, n (%)		611 (49.9)	7,601 (49.2)	7,232 (49.5)	0.67	0.80
Karnofsky score (%), n (%)	70–100	498 (40.7)	4,506 (29.2)	3,831 (26.2)	<0.001	<0.001
	40–60	547 (44.7)	6,737 (43.6)	5,812 (39.8)		
	10–30	180 (14.7)	4,202 (27.2)	4,977 (34.0)		
Recipient diabetes, n (%)		397 (32.4)	4,589 (29.7)	3,899 (26.7)	0.051	<0.001
HCV, n (%)		231 (18.9)	3,372 (21.8)	3,222 (22.0)	0.01	0.01
Alcohol related disease, n (%)		343 (28.0)	4,961 (32.1)	4,586 (31.4)	0.003	0.01
Non-alcoholic steatohepatitis, n (%)		308 (25.1)	3,042 (19.7)	2,478 (16.9)	<0.001	<0.001
HCC, n (%)		215 (17.6)	2,155 (14.0)	1,691 (11.6)	0.001	<0.001
Status 1, n (%)		12 (1.0)	269 (1.7)	442 (3.0)	0.06	<0.001
Previous liver transplant, n (%)		14 (1.1)	482 (3.1)	816 (5.6)	<0.001	<0.001
Dialysis requirement, n (%)		47 (3.8)	1,498 (9.7)	2,059 (14.1)	<0.001	<0.001
Portal thrombosis, n (%)		195 (15.9)	2,345 (15.2)	2,049 (14.0)	0.51	0.07
Mechanical ventilation, n (%)		36 (2.9)	1,170 (7.6)	1,634 (11.2)	<0.001	<0.001
Median Donor age (year), [IQR]		74 [71, 76]	53 [47, 59]	27 [22, 33]	<0.001	<0.001
Donor gender, n (%)	Male	551 (45.0)	8,519 (55.2)	9,639 (65.9)	<0.001	<0.001
	Female	674 (55.0)	6,926 (44.8)	4,981 (34.1)		
Donor race, n (%)	White	837 (68.3)	9,779 (63.3)	9,495 (64.9)	<0.001	<0.001
	Black	171 (14.0)	2,941 (19.0)	2,416 (16.5)		
	Hispanic	141 (11.5)	1,972 (12.8)	2,133 (14.6)		
	Others	76 (6.2)	753 (4.9)	576 (3.9)		
Donor BMI (kg/m^2^), n (%)	18.5–24.9	376 (30.7)	3,993 (25.9)	5,717 (39.1)	<0.001	<0.001
	25.0–29.9	427 (34.9)	5,046 (32.7)	4,593 (31.4)		
	30.0 ≤	392 (32.0)	6,141 (39.8)	3,747 (25.6)		
	<18.5	30 (2.4)	265 (1.7)	563 (3.9)		
Cold ischemia time (hours), n (%)	<6.0	739 (60.3)	8,653 (56.0)	7,838 (53.6)	<0.001	<0.001
	6.0–7.9	356 (29.1)	4,507 (29.2)	4,246 (29.0)		
	8.0 ≤	130 (10.6)	2,285 (14.8)	2,536 (17.3)		
DCD donor, n (%)		0 (0)	979 (6.3)	1,403 (9.6)	<0.001	<0.001
Donor cause of death, n (%)	Trauma	176 (14.4)	2,626 (17.0)	5,910 (40.4)	<0.001	<0.001
	Anoxia	222 (18.1)	5,532 (35.8)	6,677 (45.7)		
	CVA	814 (66.4)	6,963 (45.1)	1,611 (11.0)		
	Others	13 (1.1)	324 (2.1)	422 (2.9)		
Organ share type, n (%)	Local	751 (61.3)	10,237 (66.3)	9,387 (64.2)	<0.001	<0.001
	Regional	329 (26.9)	4,397 (28.5)	4,703 (32.2)		
	National	145 (11.8)	811 (5.3)	530 (3.6)		
Donor diabetes, n (%)		368 (30.0)	3,086 (20.0)	604 (4.1)	<0.001	<0.001
Donor history of heavy alcohol use, n (%)		106 (8.7)	3,190 (20.7)	1,936 (13.2)	<0.001	<0.001
Donor history of hypertension, n (%)		925 (75.5)	8,796 (57.0)	1,816 (12.4)	<0.001	<0.001
Donor history of myocardial infarction, n (%)		145 (11.8)	1,057 (6.8)	191 (1.3)	<0.001	<0.001
One-year graft loss, n (%)		140 (11.4)	1,435 (9.3)	1,196 (8.2)	0.01	<0.001

O vs. M: older group vs. middle-aged group.

O vs. Y: older group vs. younger group.

Abbreviations: BMI, body mass index; CVA, cerebrovascular accident; DCD, donation after circulatory death; HCV, hepatitis C virus; HCC, hepatocellular carcinoma; INR, international normalized ratio; MELD, model for end-stage liver disease; MELD-Na, model for end-stage liver disease-sodium.

Data was summarized using the median with interquartile range (IQR) for continuous variables and using percentage for discrete variables. Continuous variables were analyzed using the Mann-Whitney-U test and discrete variables were analyzed using a chi-square test.

Median recipient MELD (MELD-sodium) score was significantly lower in older donor group (18 vs. 22 [donors aged 40–69] and 24 [donors <40 years], *p* < 0.001 for both). More of recipients from older donors had HCC compared to recipients with younger donors (17.6% vs. 14.0% [40–69] and 11.6% [<40], *p* = 0.001 and *p* < 0.001, respectively) but fewer had HCV (18.9% vs. 21.8% and 22.0%, *p* = 0.01 for both). Recipients of organs from donors ≥70 were less likely to have Karnofsky scores of 10–30% (14.7% vs. 27.2% [40–69] and 34.0% [<40], *p* < 0.001 for both), to have previously received a liver transplant (1.1% vs. 3.1% [40–69] and 5.6% [<40], *p* < 0.001 for both), or to be registered as status 1 (1.0% vs. 1.7% [40–69] and 3.0% [<40], *p* = 0.06 and *p* < 0.001 respectively), were also more likely to have recipient diabetes (32.4% vs 29.7% [40–69] and 26.7% [<40], *p* = 0.051 and *p* < 0.001, respectively). Organs from the older donor group were more likely to have <6 h cold ischemia time than from other age groups (60.3% vs. 56.0% [40–69] and 53.6% [<40], *p* < 0.001 for both), to be allocated from a national organ share (11.8% vs. 5.3% [40–69] and 3.6% [<40], *p* < 0.001 for both), to have donor diabetes (30.0% vs 20.0% [40–69] and 4.1% [<40], *p* < 0.001 for both), history of hypertension (75.5% vs 57.0% [40–69] and 12.4% [<40], *p* < 0.001 for both), history of myocardial infarction (11.8% vs 6.8% [40–69] and 1.3% [<40], *p* < 0.001 for both), and to have liver biopsy (70.7% vs 52.0% [40–69] and 26.5% [<40], *p* < 0.001 for both), but those were less likely to have history of heavy alcohol use (8.7% vs 20.7% [40–69] and 13.2% [<40], *p* < 0.001 for both). There were no cases of donation after cardiac death (DCD) among recipients of organs from the ≥70 donor group (Table 1).

### Donor Age Group as a Risk Factor for One-Year Liver Graft Loss

The adjusted risk of one-year graft loss was significantly higher among recipients of organs from donors aged ≥70 years than from donors aged 40–69 years (aHR 1.30, 95%CI 1.09–1.56, *p* = 0.004) and aged <40 years (aHR 1.39, 95%CI 1.15–1.69, *p* < 0.001; Table 2).

**TABLE 2:** Comparisons of risk for 1-year graft loss between donor age groups.

	aHR	95% CI	*p* value
Ref. middle-aged donor group	1.30	1.09–1.56	0.004
Ref. younger donor group	1.39	1.15–1.69	<0.001

Abbreviations: aHR, adjusted hazard ratio.

a Hazards were adjusted by a multivariable Cox regression model for the following variables present at the time of transplantation: recipient age, recipient gender, recipient race, recipient body mass index (BMI), recipient diabetes, recipient primary liver disease etiologies including HCV, infection, alcohol related disease, non-alcoholic steatohepatitis and other diseases, hepatocellular carcinoma, international normalized ratio, serum bilirubin, serum creatinine, Karnofsky score, history of abdominal surgery, dialysis requirement, serum sodium, portal thrombus, mechanical ventilation, previous liver transplant, status 1, cold ischemia time, donation after circulatory death, donor gender, donor race, donor BMI, donor diabetes, donor cause of death, organ share type, donor history of heavy alcohol use, donor history of hypertension, and donor history of myocardial infarction.

### Risk Factor Analysis in Older Donor Group and Risk Stratification System

Demographic comparisons between the derivation and validation cohorts are shown in the Table 3. In the derivation dataset, the following recipient characteristics were associated with significantly increased risk of graft loss: previous liver transplant (aHR 4.37, 95%CI 1.98–9.65, *p* < 0.001); need for mechanical ventilation (aHR 4.28, 95%CI 1.95–9.43, *p* < 0.001); portal thrombus (aHR 1.87, 95%CI 1.26–2.77, *p* = 0.001); serum sodium <125mEq/L (aHR 2.88, 95%CI 1.34–6.20, *p* = 0.007); Karnofsky score between 10 and 30% (aHR 2.03, 95%CI 1.13–3.65, *p* = 0.01), between 40%–60% (aHR 1.65, 95%CI 1.08–2.51, *p* = 0.02; Table 4). HCV status did not increase risk of graft loss in the older donor group. Using these results, a risk stratification system was created using same (derivation) dataset by multiplying the aHRs of the significant risk factors (Table 5). Based on ROC curve analysis (Supplementary Figure S1), a risk score cut-off value of 2.03 was used to divide patients into low-risk (<2.03; *n* = 931) and high-risk groups (≥2.03; *n* = 294).

**TABLE 3:** Comparisons of characteristics between the derivation and validation cohorts.

Characteristics	Group	Derivation	Validation	*p* Value
		*n* = 31,290	*n* = 6,485	
Median recipient age (year), [IQR]		58 [50, 64]	57 [48, 64]	0.001
Recipient gender, n (%)	Male	20,753 (66.3)	4,191 (64.6)	0.009
	Female	10,537 (33.7)	2,294 (35.4)	
Recipient race, n (%)	White	22,284 (71.2)	4,575 (70.5)	0.001
	Black	2,544 (8.1)	463 (7.1)	
	Hispanic	4,642 (14.8)	1,063 (16.4)	
	Others	1,820 (5.8)	384 (5.9)	
Recipient BMI (kg/m^2^), n (%)	18.5–24.9	8.004 (25.6)	1,650 (25.4)	0.002
	25.0–29.9	10,653 (34.0)	2,067 (31.9)	
	30.0 ≤	12,173 (38.9)	2,673 (41.2)	
	<18.5	460 (1.5)	95 (1.5)	
Median MELD (MELD-Na) score, [IQR]		22 [14, 32]	25 [16, 32]	<0.001
Serum bilirubin (mg/dl), n (%)	<2.0	9,281 (29.7)	1,650 (25.4)	<0.001
	2.0–4.4	7,424 (23.7)	2,067 (31.9)	
	4.5–11.9	6,906 (22.1)	2,673 (41.2)	
	12.0 ≤	7,679 (24.5)	95 (1.5)	
INR, n (%)	<1.5	11,718 (37.4)	2,070 (31.9)	<0.001
	1.5–1.7	5,479 (17.5)	1,187 (18.3)	
	1.8–2.4	7,519 (24.0)	1,736 (26.8)	
	2.5 ≤	6,574 (21.0)	1,492 (23.0)	
Serum creatinine (mg/dl), n (%)	<1.5	22,984 (73.5)	4,570 (70.5)	<0.001
	1.5–1.7	2,447 (7.8)	560 (8.6)	
	1.8–2.5	2,976 (9.5)	664 (10.2)	
	2.6 ≤	2,883 (9.2)	691 (10.7)	
Serum sodium (mEq/L), n (%)	135–145	19.778 (63.2)	3,824 (59.0)	<0.001
	125–134	9,910 (31.7)	2,354 (36.3)	
	<125	849 (2.7)	189 (2.9)	
	146 ≤	753 (2.4)	118 (1.8)	
History of abdominal surgery, n (%)		15,444 (49.4)	3,021 (46.6)	<0.001
Karnofsky score (%), n (%)	70–100	8,835 (28.2)	1,922 (29.6)	<0.001
	40–60	13,096 (41.9)	2,537 (39.1)	
	10–30	9,359 (29.9)	2,026 (31.2)	
Recipient diabetes, n (%)		8,885 (28.4)	1,827 (28.2)	0.72
HCV, n (%)		6,825 (21.8)	1,023 (16.1)	<0.001
Alcohol related disease, n (%)		9,890 (31.6)	2,682 (41.4)	<0.001
Non-alcoholic steatohepatitis, n (%)		5,828 (18.6)	1,520 (23.4)	<0.001
HCC, n (%)		4,061 (13.0)	1,514 (23.3)	<0.001
Status 1, n (%)		723 (2.3)	0 (0)	<0.001
Previous liver transplant, n (%)		1,313 (4.2)	268 (4.1)	0.84
Dialysis requirement, n (%)		3,604 (11.5)	887 (13.7)	<0.001
Portal thrombosis, n (%)		4,589 (14.7)	859 (13.2)	0.003
Mechanical ventilation, n (%)		2,840 (9.1)	535 (8.2)	0.03
Median Donor age (year)		41 [28, 55]	41 [29, 55]	0.10
Donor age (year), n (%)	<40	14,620 (46.7)	3,011 (46.4)	0.43
	40–69	15,445 (49.4)	3,239 (49.9)	
	70 ≤	1,225 (3.9)	235 (3.6)	
Donor gender, n (%)	Male	18,709 (59.8)	3,962 (61.1)	0.053
	Female	12,581 (40.2)	2,523 (38.9)	
Donor race, n (%)	White	20.111 (64.3)	4,146 (63.9)	0.08
	Black	5,528 (17.7)	1,155 (17.8)	
	Hispanic	4,246 (13.6)	930 (14.3)	
	Others	1,405 (4.5)	254 (3.9)	
Donor BMI (kg/m^2^), n (%)	18.5–24.9	10,086 (32.2)	1,996 (30.8)	0.055
	25.0–29.9	10.066 (32.2)	2,086 (32.2)	
	30.0 ≤	10,280 (32.9)	2,230 (34.4)	
	<18.5	858 (2.7)	171 (2.6)	
Cold ischemia time (hours), n (%)	<6.0	17,230 (55.1)	3,613 (55.7)	<0.001
	6.0–7.9	9,109 (29.1)	2,048 (31.6)	
	8.0 ≤	4,951 (15.8)	824 (12.7)	
DCD donor, n (%)		2,382 (7.6)	697 (10.7)	<0.001
Donor cause of death, n (%)	Trauma	8,712 (27.8)	1,640 (25.3)	<0.001
	Anoxia	12,431 (39.7)	2,948 (45.5)	
	CVA	9,388 (30.0)	1,752 (27.0)	
	Others	759 (2.4)	145 (2.2)	
Organ share type, n (%)	Local	20,375 (65.1)	2,553 (39.4)	<0.001
	Regional	9,429 (30.1)	2,022 (31.2)	
	National	1,486 (4.7)	1,910 (29.5)	
Donor diabetes, n (%)		4,058 (13.0)	867 (13.4)	0.39
Donor history of heavy alcohol use, n (%)		5,232 (16.7)	1,176 (18.1)	0.006
Donor history of hypertension, n (%)		11,537 (36.9)	2,418 (37.3)	0.53
Donor history of myocardial infarction, n (%)		1,393 (4.5)	336 (5.2)	0.01
One-year graft loss, n (%)		2,771 (8.9)	573 (8.8)	0.97

Abbreviations: BMI, body mass index; CVA, cerebrovascular accident; DCD, donation after circulatory death; HCV, hepatitis C virus; HCC, hepatocellular carcinoma; INR, international normalized ratio; MELD, model for end-stage liver disease; MELD-Na, model for end-stage liver disease-sodium.

Data was summarized using the median with interquartile range (IQR) for continuous variables and using percentage for discrete variables. Continuous variables were analyzed using the Mann-Whitney-U test and discrete variables were analyzed using a chi-square test.

**TABLE 4:** Risk for 1-year graft loss after liver transplantation in older donor derivation group.

Factors	aHR	95% CI	*p* value
Previous liver transplant	4.37	1.98–9.65	<0.001
Mechanical ventilation	4.28	1.95–9.43	<0.001
Portal thrombus	1.87	1.26–2.77	0.001
Serum sodium <125 mEq/L [ref. 135–145 mEq/L]	2.88	1.34–6.20	0.007
Serum sodium 125–135 mEq/L [ref. 135–145 mEq/L]	1.35	0.92–1.99	0.13
Serum sodium 146 mEq/L or higher [ref. 135–145 mEq/L]	1.36	0.51–3.65	0.54
Karnofsky score 10–30% [ref. 70–100%]	2.03	1.13–3.65	0.01
Karnofsky score 40–60% [ref. 70–100%]	1.65	1.08–2.51	0.02
Cold ischemia time 8 h or longer [ref. < 6.0 h]	1.67	1.05–2.65	0.03
Cold ischemia time 6.0–7.9 h [ref. < 6.0 h]	0.86	0.57–1.30	0.47
Serum bilirubin 12 mg/dl or higher [ref. < 2.0 mg/dl]	0.82	0.39–1.72	0.60
Serum bilirubin 4.5–11.9 mg/dl [ref. < 2.0 mg/dl]	0.85	0.47–1.55	0.60
Serum bilirubin 2.0–4.4 mg/dl [ref. < 2.0 mg/dl]	1.29	0.84–1.98	0.25
INR 2.5 or higher [ref. < 1.5]	1.03	0.56–1.88	0.93
INR 1.8–2.4 [ref. < 1.5]	0.59	0.34–1.01	0.056
INR 1.5–1.7 [ref. < 1.5]	0.72	0.45–1.17	0.18
Serum creatinine 2.6 mg/dl or higher [ref. < 1.5 mg/dl]	1.91	0.95–3.82	0.07
Serum creatinine 1.8–2.5 mg/dl [ref. < 1.5 mg/dl]	0.72	0.38–1.38	0.32
Serum creatinine 1.5–1.7 mg/dl [ref. < 1.5 mg/dl]	0.96	0.51–1.79	0.89
HCV positive	0.68	0.41–1.12	0.13
Status 1	0.62	0.16–2.45	0.50
Dialysis requirement	0.64	0.27–1.50	0.30
Donor BMI 30 kg/m^2^ or higher [ref. 18.5–24.9 kg/m^2^]	0.93	0.61–1.44	0.76
Donor BMI 25.0–29.9 kg/m^2^ [ref. 18.5–24.9 kg/m^2^]	1.07	0.70–1.63	0.77
Donor BMI <18.5 kg/m^2^ [ref. 18.5–24.9 kg/m^2^]	1.49	0.60–3.69	0.39

Abbreviations: aHR, adjusted hazard ratio; BMI, body mass index; HCV, hepatitis C virus; INR, international normalized ratio.

a Hazards were adjusted by a multivariable Cox regression model for the following variables present at the time of transplantation: hepatitis C virus, international normalized ratio, serum bilirubin, serum creatinine, Karnofsky score, dialysis requirement, serum sodium, portal thrombus, mechanical ventilation, previous liver transplant, status 1, cold ischemia time, and donor BMI.

**TABLE 5:** Assigned risk score points and categorization of risk groups in older donor group.

Risk factors	aHR
Previous liver transplant	4.37
Mechanical ventilation	4.28
Portal thrombus	1.87
Serum sodium <125 mEq/L [ref. 135–145 mEq/L]	2.88
Karnofsky score 10–30% [ref. 70–100%]	2.03
Karnofsky score 40–60% [ref. 70–100%]	1.65

aHR, adjusted hazard ratio.

Although patient age was not a significant risk factor for graft loss among recipients in the older donor group, age ≥65 years was a significant risk factor for graft loss among recipients who received organs from the middle-aged or younger donors (aHR 1.19, 95%CI 1.01–1.40 and aHR 1.71, 95%CI 1.44–2.04; *p* = 0.04 and *p* < 0.001, respectively). Recipient age of 50–64%years was also a significant risk factor for graft loss in the younger donor group (aHR 1.27, 95%CI 1.09–1.47, *p* = 0.001; Supplementary Tables S1, S2).

### Comparison of 1-Year Risk of Graft Loss in the Older Donor Group Using the Risk Score System

One-year graft survival rate was significantly higher in low-risk recipients than in high-risk recipients (91.7% [low-risk] vs. 78.0% [high-risk], *p* < 0.001) (Supplementary Figure S2A). Three- and 5-year graft survival rate were also significantly higher in low-risk recipients than in high-risk recipients (3-year; 82.5% [low-risk] vs. 70.5% [high-risk], *p* < 0.001, 5-year; 76.0% [low-risk] vs. 64.1% [high-risk], *p* < 0.001) (Supplementary Figures S2B,C). One-year graft survival rate in low-risk recipients of the older donor group was similar to the younger or middle-aged donor group (Figure 2A). After excluding DCD cases from the middle-aged and younger donor groups (for consistency with the older donor group, in which there was no DCD donors), similar trends were observed (Figure 2B).

The adjusted risk of one-year graft loss in the low-risk older donor group was similar to that of the younger donor group (aHR 1.08, 95% CI 0.85–1.38, *p* = 0.53; Figure 2C **)**. In contrast, the adjusted risk of one-year graft loss in the high-risk recipients was significantly higher than in the younger donor group (aHR 2.00, 95% CI 1.53–2.61, *p* < 0.001). While the adjusted risk of one-year graft loss in the high-risk recipients was also significantly higher compared to the middle-aged donor group (aHR 1.84, 95% CI 1.42–2.38, *p* < 0.001), those in the low-risk older donor group was similar to that of the middle-aged donor group (aHR 0.99, 95% CI 0.78–1.26, *p* = 0.95; Figure 2D).

### Comparison of Risk for Graft Loss in the Older Donor Using the Validation Dataset

**FIGURE 3:**
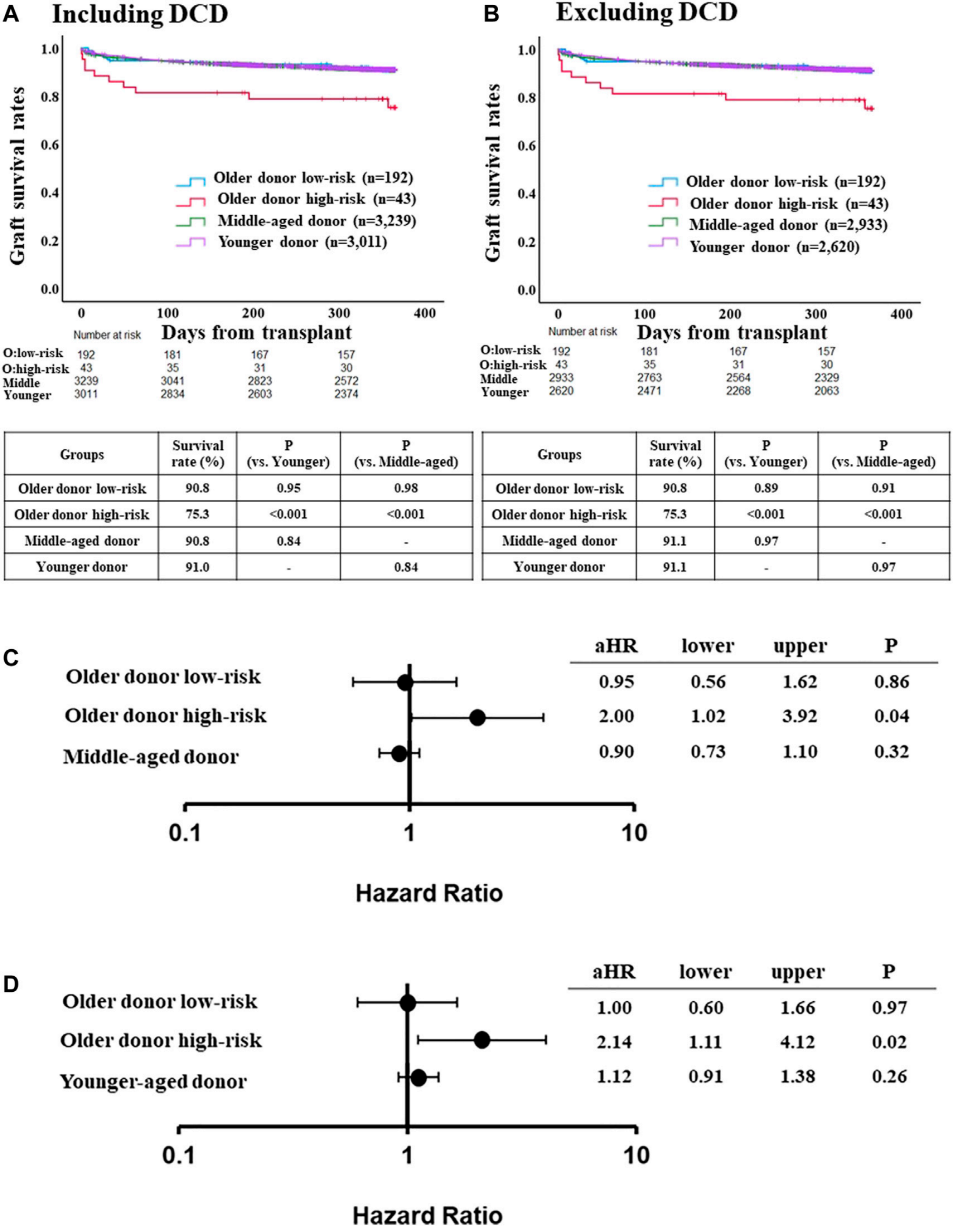
Comparison of post-LT outcome between the older donor validation group stratified by the risk classification and middle-aged or younger donor group. **(A)** One-year graft survival rate in low-risk recipients of the older donor group was similar to those in middle-aged or younger donor group (*p* = 0.98, *p* = 0.95, respectively). **(B)** One-year graft survival rate in low-risk recipients of the older donor group was similar to those in middle-aged or younger donor group after excluding DCD cases (*p* = 0.91, *p* = 0.89, respectively). **(C)** Adjusted hazards of graft loss in the older donor validation group stratified by the risk classification and middle-aged donor group (ref. younger donor group). **(D)** ref. middle-aged donor group. Hazards were adjusted by a multivariable Cox regression model for the following variables present at the time of transplantation: recipient age; recipient gender; recipient race; recipient body mass index (BMI); recipient diabetes; recipient primary liver disease etiologies including HCV infection, alcohol related disease, non-alcoholic steatohepatitis and other diseases; hepatocellular carcinoma; international normalized ratio; serum bilirubin; serum creatinine; Karnofsky score; history of abdominal surgery; dialysis requirement; serum sodium; portal thrombus; mechanical ventilation; previous liver transplant; cold ischemia time; donor gender; donor race; donor BMI; donor diabetes; donor cause of death; organ share type; donor history of heavy alcohol use; donor history of hypertension; and donor history of myocardial infarction.

Among the validation cohort, one-year graft survival rate in low-risk recipients of the older donor group was similar to those in the younger or middle-aged donor group (Figure 3A). After excluding DCD cases from the younger and middle-aged donor groups, the one-year graft survival rate in low-risk recipients of the older donor group was similar to those in the younger or middle-aged donor group (Figure 3B).

The adjusted risk of one-year graft loss was similar between low-risk older donor recipients and younger donor recipients (aHR 0.95, 95% CI 0.56–1.62, *p* = 0.86; Figure 3C), but was significantly higher for high-risk recipients (aHR 2.00, 95% CI 1.02–3.92, *p* = 0.04). While the adjusted risk of one-year graft loss in the high-risk recipients was also significantly higher compared to the middle-aged donor group (aHR 2.14, 95% CI 1.11–4.12, *p* = 0.02), those in the low-risk older donor group was similar to that of the middle-aged donor group (aHR 1.00, 95% CI 0.60–1.66, *p* = 0.97; Figure 3D).

## Discussion

Using a systematic approach to identify risk factors for graft loss among recipients of liver transplant from donors ≥70 years old, we were able to categorize patients into low- and high-risk groups. In general, the recipients of organs from older donors at highest risk of one-year graft loss had multiple risk factors—including previous liver transplant, mechanical ventilation, portal thrombus, low serum sodium value, and low Karnofsky score—that indicated they were often considerably more ill at the time of transplantation, compared to others. With regard to laboratory values associated with MELD-sodium score, serum sodium was considered as a significant risk factor, but not total bilirubin, INR, or serum creatinine. As expected, donor age of 70 years or older was found to be a risk factor for one-year graft loss. However, according to our risk stratification system, low-risk recipients of organs from older donors had similar outcomes to those of recipients from younger and middle-aged donor groups. We further evaluated our risk stratification system in a separate validation dataset with consistent results, confirming its applicability. These findings indicate that, while advanced donor age may be a risk factor for negative post-LT outcomes, organs from older donors can be safely used with careful recipient selection, which might help expand donor pool without compromising LT outcomes.

A strength of our approach was adjustment for both recipient and donor characteristics. Although donor and organ characteristics such as race, BMI, cold ischemia time, and donor location have been shown to be associated with post-LT outcomes [3, 19], there were no significant donor characteristics other than prolonged CIT among the risk factors for one-year graft loss in our sample of recipients of organs from donors ≥70 years. While DCD donor is usually considered as a donor risk factor associated with poor post-LT outcomes, there was no DCD donor in this older donor group (≥70 years). Therefore, the prognostic impact of these donor characteristics in the older donor group could not be assessed in this study. It should be noted that possible risks associated with these factors should not be ignored when using older donors. However, we acknowledge that no stratification system should be considered “one-size-fits-all,” and that it remains important to carefully assess donor characteristics when using liver grafts from older donors. Of note, in our between-group comparisons of graft loss among recipients of organs from younger, middle-aged, and low-risk/older donors, analyses were adjusted for a number of donor characteristics that are known risk factors for graft loss.

According to a previous report by Haugen et al. [20], outcomes among recipients of liver grafts from donors ≥70 years have improved over time, with a 40% reduction in risk of graft loss in 2010–2016 versus 2003–2009; however, rates of graft loss are still higher than with grafts from donors <70 years. In our analysis of more recent data (2014–2019) we found that donor age ≥70 years remains a significant risk factor for graft loss. Notably, the proportion of donors aged ≥70 in Haugen’s report—3.2% of all recipients—is consistent with our own [20]. Although this is a relatively small number, confidence in the safety of liver grafts from older donors could lead to expansion of the donor pool.

In our risk-stratification system, low-risk recipients of livers from older donors accounted for 76.0% of patients who received from donors of 70 years or older. Also, post-LT outcomes in these patients were similar to those of recipients with organs from younger (<40 years) and middle-aged donors (40–69 years). These results suggest that careful recipient selection may reduce risks associated with using old donors, which might decrease organ discard rate and expand the donor pool safely. A number of previous reports have focused on preferred recipient characteristics for grafts from elderly donors [8–10]. [9] suggested that preferred patient profile for using grafts from donors ≥70 years were being a first-time recipient over the age of 45, with BMI <35, non-status 1 registration, cold ischemic time <8 h, and either a non-HCV indication for transplant or hepatocellular carcinoma [9]. According to a French study, elderly grafts (age >75) may be safely used if donation occurred after brain death and recipients were HCV negative and had not previous undergone transplantation [21]. Previous liver transplant has been commonly reported as a strong risk factor for poor post-LT outcomes, which is consistent with our results. In contrast, although previous studies have indicated that grafts from older donors may lead to worse post-LT outcomes in patients with HCV [8–10], we did not observe the same impact of HCV-positive status on negative outcomes. At least one study has found that direct-acting antiviral treatments allowed a safe use of liver grafts from donors >70 years in HCV-positive recipients [22]. Given that our study included only patients transplanted after 2014, when direct-acting antiviral therapy became widely available, this may explain why HCV was no longer a significant risk factor in our results.

Advanced recipient age is also a known risk factor for liver graft loss [3, 4]. However, we did not find recipient age to be significantly associated with loss of grafts in the older donor group, but it was a risk factor in recipients of organs from the middle-aged and younger donor groups. Other studies have reported conflicting results regarding recipient-donor age matching. Bittermann et al. reported that in younger recipients (<40 years), the risk of graft failure increased with donor age, but that risk of loss in grafts from older donors (≥60 years) were similar regardless of recipient age [23]. Likewise, Chapman et al, reported comparable outcomes in graft and patient survival with older donors (≥60 years old), without an increased rate of complications, regardless of recipient age [24]. Our results concurred with the above results. While the use of older donor liver grafts might achieve satisfactory post-LT outcomes regardless of recipient age, the possibility of increased risk with increased recipient age should be acknowledged.

In the past, many transplant centers would not accept DCD donors older than 60 years old, as there were reports of higher risk of graft loss with older DCD donors [25-27]. More recent studies have suggested that selected grafts from elderly DCD donors could achieve an acceptable graft survival rate [28, 29]. In our study, there were no DCD grafts in patients who received grafts from donors 70 years or older, and thus we could not evaluate the impact of DCD grafts on recipients from elderly donors. Recently, the utility of normothermic perfusion for DCD grafts has been reported [30, 31]. Normothermic perfusion has proven its beneficial effect on ischemia-reperfusion injury, which could potentially lead to improved post-LT outcomes, when using older DCD donors. Although there was no report about normothermic perfusion for older DCD grafts, it may be a promising strategy. Czigany et al. reported that among patients who received extended criteria liver (median donor age 72 years old) from donation after brain death grafts, hypothermic oxygenated machine perfusion reduced early allograft injury and improved post-transplant outcomes by multicenter randomized controlled trial [32].

There are a number of limitations to our analysis. This is a retrospective study using the OPTN/UNOS registry, which lacks detailed post-transplant clinical data, such as surgical complications after transplantation. We were also limited by the small proportion of donors ≥70 years in the dataset. Although we were able to detect a number of significant risk factors despite the relative small sample size, it is possible that a larger sample size would have provided more precision in our estimates. The primary outcome examined in this study (one-year graft loss) was a short-term outcome and may not be applicable to mid-to long-term outcomes. Three-year and 5-year graft survival were evaluated, which demonstrated that the negative impact of recipient risk factors was more prominent in the first year post-transplant, then the survival curves became parallel after 1 year between the low and high risk groups. Also, we could not evaluate the impact of grafts after DCD in elderly donors due to the absence of such donors in the dataset. Despite these limitations, the scoring system could be useful to determine suitable recipient selection when using the liver graft from older donors. Our scoring system would not be used to regulate organ acceptance practice. Transplant physicians and centers could use it to estimate its risk and should decide indications at their discretion if those risks are acceptable for each case.

In conclusion, our risk stratification system using the following recipient factors, history of the previous liver transplant, low Karnofsky Performance Status score, need for mechanical ventilation, presence of portal vein thrombosis, and hyponatremia, might be useful for recipient selection who are eligible for liver grafts from older donors. This could lead to further expansion of the donor pool without compromising outcomes.

## Data Availability

The data that support the findings of this study are available from Organ Procurement and Transplantation Network (OPTN). Restrictions apply to the availability of these data, which were used under license for this study. Data are available from OPTN at https://optn.transplant.hrsa.gov/data/request-data/with the permission of OPTN and United Network of Organ Sharing (UNOS).

## Supplementary Material

Supplementary Figure S1 ROC curve between a risk score and one-year graft survival.

Supplementary Figure S2 Comparison of post-LT outcome between the older donor low and high-risk groups stratified by the risk classification. One-year graft survival rate in low-risk recipients of the older donor group was significantly higher than those in high-risk recipients (91.7% vs. 78.0%, *p* < 0.001). **(B)** Three-year graft survival rate in low-risk recipients of the older donor group was significantly higher than those in high-risk recipients (82.5% vs. 70.5%, *p* < 0.001). **(C)** Five-year graft survival rate in low-risk recipients of the older donor group was significantly higher than those in high-risk recipients (76.0% vs. 64.1%, *p* < 0.001).

## Abbreviations

aHR, adjusted hazard ratio; BAR, balance of risk; BMI, body mass index; CVA, cerebrovascular accident; DCD, donation after circulatory death; HCV, hepatitis C virus; HCC, hepatocellular carcinoma; INR, international normalized ratio; IQR, interquartile range; LT, liver transplant; MELD, model for end-stage liver disease; OPTN, organ procurement and transplant network; ROC, receiver operating characteristic; STAR, standard transplant and research; UNOS, united network for organ sharing.
