# Supplementary figures and images for: Liver Transplant Recipient Characteristics Associated With Worse Post-Transplant Outcomes in Using Elderly Donors
Source: Transpl Int. 2022 Aug 25;35:10489. doi: 10.3389/ti.2022.10489 (PMC9452632; doi:10.3389/ti.2022.10489)

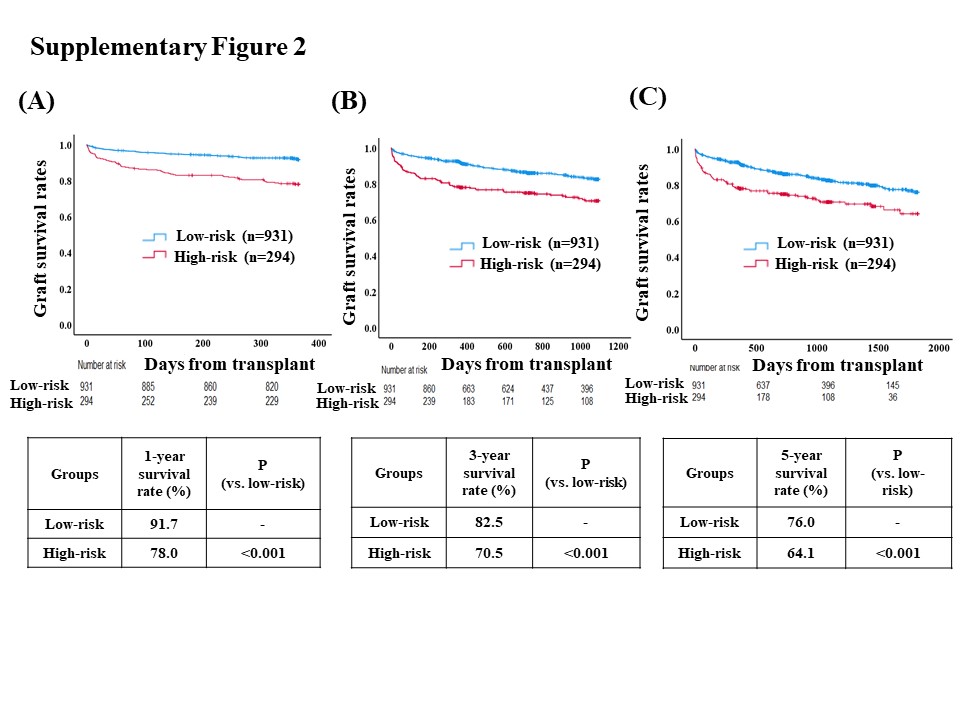

Supplement: Supplementary file 1 [file Image2.jpg]

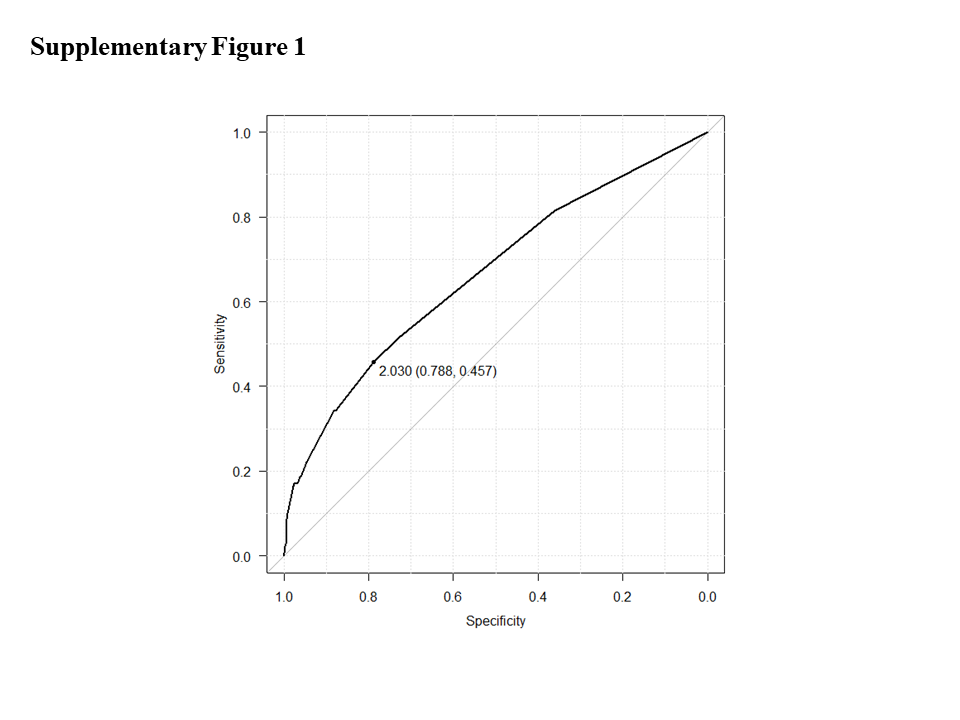

Supplement: Supplementary file 2 [file Image1.TIF]
