# Supplementary material for: Liver Transplant Recipient Characteristics Associated With Worse Post-Transplant Outcomes in Using Elderly Donors
Source: Transpl Int. 2022 Aug 25;35:10489. doi: 10.3389/ti.2022.10489 (PMC9452632; doi:10.3389/ti.2022.10489)
Supplement: Supplementary file 3 [file DataSheet1.docx]

| **Supplementary Table 1. Risk for 1-year graft loss after liver transplantation in middle-aged donor group** | | | | |
| --- | --- | --- | --- | --- |
| Factors | aHR | 95% CI | P value |  |
| Previous liver transplant | 1.86 | 1.48-2.33 | <0.001 |  |
| Mechanical ventilation | 1.41 | 1.15-1.72 | <0.001 |  |
| Portal thrombus | 1.30 | 1.14-1.49 | <0.001 |  |
| Serum sodium < 125 mEq/L [ref. 135-145 mEq/L] | 0.91 | 0.64-1.29 | 0.58 |  |
| Serum sodium 125-135 mEq/L [ref. 135-145 mEq/L] | 0.95 | 0.84-1.07 | 0.43 |  |
| Serum sodium 146 mEq/L or higher [ref. 135-145 mEq/L] | 1.46 | 1.11-1.91 | 0.006 |  |
| Karnofsky score 10-30% [ref. 70-100%] | 1.55 | 1.29-1.86 | <0.001 |  |
| Karnofsky score 40-60% [ref. 70-100%] | 1.19 | 1.04-1.37 | 0.01 |  |
| Recipient age 65 yo or older [ref. < 50 yo] | 1.19 | 1.01-1.40 | 0.04 |  |
| Recipient age 50-64 yo [ref. < 50 yo] | 1.10 | 0.95-1.26 | 0.20 |  |
| Recipient race black [ref. white] | 1.34 | 1.13-1.60 | 0.001 |  |
| Recipient race hispanic [ref. white] | 0.96 | 0.83-1.12 | 0.64 |  |
| Recipient race others [ref. white] | 0.92 | 0.72-1.18 | 0.51 |  |
| Recipient BMI 30 kg/m^2^ or higher [ref. 18.5-24.9 kg/m^2^] | 1.06 | 0.93-1.22 | 0.38 |  |
| Recipient BMI 25.0-29.9kg/m^2^ [ref. 18.5-24.9 kg/m^2^] | 0.93 | 0.80-1.07 | 0.29 |  |
| Recipient BMI < 18.5 kg/m^2^ [ref. 18.5-24.9 kg/m^2^] | 1.28 | 0.86-1.91 | 0.23 |  |
| Serum bilirubin 12 mg/dL or higher [ref. < 2.0 mg/dL] | 0.91 | 0.75-1.12 | 0.39 |  |
| Serum bilirubin 4.5-11.9 mg/dL [ref. < 2.0 mg/dL] | 1.02 | 0.85-1.22 | 0.87 |  |
| Serum bilirubin 2.0-4.4 mg/dL [ref. < 2.0 mg/dL] | 0.99 | 0.85-1.176 | 0.95 |  |
| INR 2.5 or higher [ref. < 1.5] | 0.89 | 0.74-1.07 | 0.22 |  |
| INR 1.8-2.4 [ref. < 1.5] | 0.93 | 0.79-1.10 | 0.38 |  |
| INR 1.5-1.7 [ref. < 1.5] | 1.05 | 0.89-1.23 | 0.57 |  |
| Serum creatinine 2.6 mg/dL or higher [ref. < 1.5 mg/dL] | 1.16 | 0.96-1.39 | 0.12 |  |
| Serum creatinine 1.8-2.5 mg/dL [ref. < 1.5 mg/dL] | 1.22 | 1.02-1.45 | 0.02 |  |
| Serum creatinine 1.5-1.7 mg/dL [ref. < 1.5 mg/dL] | 1.17 | 0.97-1.40 | 0.10 |  |
| History of abdominal surgery | 1.03 | 0.93-1.15 | 0.56 |  |
| Recipient diabetes | 1.16 | 1.03-1.30 | 0.01 |  |
| Alcohol related disease | 0.83 | 0.74-0.94 | 0.004 |  |
| HCC | 0.93 | 0.78-1.10 | 0.41 |  |
| Status 1 | 0.89 | 0.62-1.26 | 0.50 |  |
| Dialysis requirement | 1.26 | 1.05-1.52 | 0.01 |  |
| Donor race black [ref. white] | 1.04 | 0.90-1.19 | 0.59 |  |
| Donor race hispanic [ref. white] | 0.98 | 0.83-1.15 | 0.76 |  |
| Donor race others [ref. white] | 1.15 | 0.91-1.44 | 0.25 |  |
| Donor BMI 30 kg/m^2^ or higher [ref. 18.5-24.9 kg/m^2^] | 1.06 | 0.92-1.22 | 0.41 |  |
| Donor BMI 25.0-29.9kg/m^2^ [ref. 18.5-24.9 kg/m^2^] | 1.18 | 1.02-1.35 | 0.02 |  |
| Donor BMI < 18.5 kg/m^2^ [ref. 18.5-24.9 kg/m^2^] | 1.48 | 1.03-2.13 | 0.03 |  |
| Cold ischemia time 8 hours or longer [ref. < 6.0 hours] | 1.54 | 1.33-1.77 | <0.001 |  |
| Cold ischemia time 6.0-7.9 hours [ref. < 6.0 hours] | 1.19 | 1.06-1.35 | 0.004 |  |
| Donor cause of death Anoxia [ref. Trauma] | 0.87 | 0.75-1.02 | 0.09 |  |
| Donor cause of death CVA [ref. Trauma] | 1.04 | 0.89-1.21 | 0.61 |  |
| Donor cause of death Others [ref. Trauma] | 0.70 | 0.45-1.10 | 0.12 |  |
| Organ share type National [ref. Local] | 1.14 | 0.90-1.43 | 0.27 |  |
| Organ share type Regional [ref. Local] | 1.00 | 0.89-1.13 | 0.99 |  |
| Donor diabetes | 1.19 | 1.04-1.36 | 0.009 |  |
| Donor history of heavy alcohol use | 0.94 | 0.82-1.07 | 0.33 |  |
| Donor history of hypertension | 1.13 | 1.00-1.27 | 0.04 |  |

**Abbreviations:** aHR, adjusted hazard ratio; BMI, body mass index; CVA, cerebrovascular accident; HCC, hepatocellular carcinoma; INR, international normalized ratio.

* Hazards were adjusted by a multivariable Cox regression model for the following variables present at the time of transplantation: recipient age, recipient race, recipient body mass index (BMI), recipient diabetes, alcohol related disease, hepatocellular carcinoma, international normalized ratio, serum bilirubin, serum creatinine, Karnofsky score, history of abdominal surgery, dialysis requirement, serum sodium, portal thrombus, mechanical ventilation, previous liver transplant, status 1, cold ischemia time, donor race, donor BMI, donor cause of death, organ share type, donor diabetes, donor history of heavy alcohol use, and donor history of hypertension.

| **Supplementary Table 2. Risk for 1-year graft loss after liver transplantation in younger donor group** | | | | |
| --- | --- | --- | --- | --- |
| Factors | aHR | 95% CI | P value |  |
| Previous liver transplant | 1.67 | 1.36-2.06 | <0.001 |  |
| Mechanical ventilation | 1.49 | 1.23-1.81 | <0.001 |  |
| Portal thrombus | 1.45 | 1.26-1.68 | <0.001 |  |
| Serum sodium < 125 mEq/L [ref. 135-145 mEq/L] | 1.22 | 1.84-1.75 | 0.30 |  |
| Serum sodium 125-135 mEq/L [ref. 135-145 mEq/L] | 0.95 | 0.83-1.08 | 0.43 |  |
| Serum sodium 146 mEq/L or higher [ref. 135-145 mEq/L] | 1.55 | 1.17-2.04 | 0.002 |  |
| Karnofsky score 10-30% [ref. 70-100%] | 1.32 | 1.08-1.62 | 0.006 |  |
| Karnofsky score 40-60% [ref. 70-100%] | 1.17 | 0.99-1.38 | 0.06 |  |
| Recipient age 65 yo or older [ref. < 50 yo] | 1.71 | 1.44-2.04 | <0.001 |  |
| Recipient age 50-64 yo [ref. < 50 yo] | 1.27 | 1.09-1.47 | 0.001 |  |
| Recipient race black [ref. white] | 1.30 | 1.08-1.58 | 0.006 |  |
| Recipient race hispanic [ref. white] | 0.92 | 0.77-1.08 | 0.31 |  |
| Recipient race others [ref. white] | 1.21 | 0.97-1.52 | 0.09 |  |
| Serum bilirubin 12 mg/dL or higher [ref. < 2.0 mg/dL] | 1.17 | 0.94-1.46 | 0.16 |  |
| Serum bilirubin 4.5-11.9 mg/dL [ref. < 2.0 mg/dL] | 1.08 | 0.87-1.34 | 0.48 |  |
| Serum bilirubin 2.0-4.4 mg/dL [ref. < 2.0 mg/dL] | 1.08 | 0.89-1.30 | 0.43 |  |
| INR 2.5 or higher [ref. < 1.5] | 0.97 | 0.79-1.19 | 0.77 |  |
| INR 1.8-2.4 [ref. < 1.5] | 1.02 | 0.84-1.23 | 0.85 |  |
| INR 1.5-1.7 [ref. < 1.5] | 1.04 | 0.86-1.26 | 0.68 |  |
| Serum creatinine 3.0 mg/dL or higher [ref. < 1.1 mg/dL] | 1.14 | 0.94-1.38 | 0.17 |  |
| Serum creatinine 2.0-2.9 mg/dL [ref. < 1.1 mg/dL] | 1.19 | 0.99-1.42 | 0.06 |  |
| Serum creatinine 1.1-1.9 mg/dL [ref. < 1.1 mg/dL] | 1.14 | 0.93-1.40 | 0.21 |  |
| History of abdominal surgery | 1.11 | 0.99-1.26 | 0.08 |  |
| Recipient diabetes | 1.08 | 0.94-1.23 | 0.27 |  |
| HCV | 0.91 | 0.77-1.08 | 0.29 |  |
| Alcohol related disease | 0.78 | 0.67-0.90 | 0.001 |  |
| Non-alcoholic steatohepatitis | 0.99 | 0.83-1.18 | 0.92 |  |
| HCC | 0.98 | 0.80-1.20 | 0.82 |  |
| Status 1 | 0.97 | 0.72-1.31 | 0.86 |  |
| Dialysis requirement | 1.19 | 0.99-1.43 | 0.06 |  |
| Donor gender male | 0.96 | 0.85-1.09 | 0.57 |  |
| Cold ischemia time 8 hours or longer [ref. < 6.0 hours] | 1.35 | 1.16-1.57 | <0.001 |  |
| Cold ischemia time 6.0-7.9 hours [ref. < 6.0 hours] | 1.06 | 0.93-1.21 | 0.40 |  |
| Donor cause of death Anoxia [ref. Trauma] | 1.07 | 0.94-1.22 | 0.28 |  |
| Donor cause of death CVA [ref. Trauma] | 1.35 | 1.12-1.64 | 0.001 |  |
| Donor cause of death Others [ref. Trauma] | 1.20 | 0.86-1.67 | 0.28 |  |
| Organ share type National [ref. Local] | 1.16 | 0.85-1.57 | 0.36 |  |
| Organ share type Regional [ref. Local] | 1.06 | 0.93-1.20 | 0.41 |  |
| Donor diabetes | 1.26 | 0.97-1.64 | 0.08 |  |
| Donor history of heavy alcohol use | 0.88 | 0.74-1.05 | 0.17 |  |
| Donor history of hypertension | 1.07 | 0.90-1.28 | 0.44 |  |

**Abbreviations:** aHR, adjusted hazard ratio; CVA, cerebrovascular accident; HCV, hepatitis C virus; HCC, hepatocellular carcinoma; INR, international normalized ratio.

* Hazards were adjusted by a multivariable Cox regression model for the following variables present at the time of transplantation: recipient age, recipient race, recipient diabetes, HCV infection, alcohol related disease, non-alcoholic steatohepatitis, hepatocellular carcinoma, international normalized ratio, serum bilirubin, serum creatinine, Karnofsky score, history of abdominal surgery, dialysis requirement, serum sodium, portal thrombus, mechanical ventilation, previous liver transplant, status 1, cold ischemia time, donor gender, donor cause of death, organ share type, donor diabetes, donor history of heavy alcohol use, and donor history of hypertension.
